# Muscle Mass Assessment in Sarcopenia: A Narrative Review

**DOI:** 10.31662/jmaj.2023-0053

**Published:** 2023-09-29

**Authors:** Isao Muraki

**Affiliations:** 1 Public Health, Osaka University Graduate School of Medicine, Suita, Japan

**Keywords:** sarcopenia, muscle mass, measurement

## Abstract

Sarcopenia is a condition characterized by age-related muscle loss and dysfunction. Over the past decade, several working groups have developed diagnostic criteria for sarcopenia, including muscle mass, grip strength, and gait speed measurements. However, there is debate over which muscle mass indicator is the most appropriate. Some groups used appendicular lean mass divided by height squared, whereas others used appendicular lean mass divided by body mass index. In addition, the association between muscle mass and long-term health outcomes is inconsistent. As a result, some experts question the necessity of using muscle mass as a diagnostic criterion for sarcopenia. This review summarizes the measurement methods and muscle mass indicators of previous studies, highlighting issues with past muscle mass assessments.

## Introduction

Three decades ago, sarcopenia was first conceptualized as age-related muscle loss and dysfunction ^(1)^. Since 2010, sarcopenia has been defined as the combination of muscle mass, grip power, and gait speed by the European Society for Clinical Nutrition and Metabolism (ESPEN) Special Interest Group ^(2)^ and the European Working Group on Sarcopenia in Older People (EWGSOP) ^(3)^. The definition by the Asian Working Group for Sarcopenia was determined 5 years later, according to the EWGSOP definition ^(4)^. Although the definitions by EWGSOP and the Asian Working Group for Sarcopenia included skeletal muscle mass index (SMI) which was calculated as appendicular lean mass (ALM) divided by height squared ^(3), (4)^, the Foundation for the National Institutes of Health (FNIH) sarcopenia project adopted ALM divided by body mass index (BMI) instead of SMI ^(5)^. In the last decade, many sarcopenia working groups have developed and updated the diagnosis criteria for sarcopenia (Table 1) ^(2), (3), (4), (5), (6), (7), (8), (9), (10), (11)^. However, the best muscle mass indicator remains unknown, possibly due to inconsistency in the association with long-term health outcomes, including physical function and mortality. Furthermore, the recent criteria for sarcopenia have shifted toward dynapenia by omitting muscle mass criteria from essential sarcopenia criteria ^(11)^. During the process of achieving consensus on the recent criteria of the Sarcopenia Definition and Outcomes Consortium, expert opinions regarding whether muscle mass is essential for diagnosing sarcopenia have been controversial.

**Table 1.** Definition of Sarcopenia.

Consensus group	Year	A. Muscle mass	B. Muscle strength	C. Physical performance	Diagnosis
ESPEN-SIG ^(2)^	2010	ALM/weight ≤ −2 SD of 18-39 years in the same sex and ethnicity	-	Gait speed <0.8 m/s in 4-m walk	C and A
EWGSOP ^(3)^	2010	ALM/height^2^ (kg/m^2^)	Grip strength (kg)	Gait speed ≤0.8 m/s	(B and/or C) and A
		≤5.67 (F), ≤7.23 (M)	<20 (F), <30 (M)		
IWGS ^(6)^	2011	ALM/height^2^ (kg/m^2^)	-	Gait speed ≤1.0 m/s	C and A
		≤5.67 (F), ≤7.23 (M)			
SCWD ^(7)^	2011	ALM/height^2^ ≤ −2 SD of 20 and 30 years in the same ethnicity	-	Gait speed ≤1.0 m/s	C and A
				400-m walk ≥6 min or fail	
AWGS ^(4)^	2014	ALM/height^2^ (kg/m^2^)	Grip strength (kg)	Gait speed <0.8 m/s in 6-m walk	(B and/or C) and A
		DEXA: ≤5.40 (F), ≤7.00 (M)	<18 (F), <26 (M)		
		BIA: ≤5.70 (F), ≤7.00 (M)			
FNIH ^(5)^	2014	ALM/BMI (m^2^)	Grip strength (kg)	-	B and A
		≤0.512 (F), ≤0.789 (M)	<16 (F), <26 (M)		
EWGSOP ^(9)^	2018	ALM (kg) <15 (F), <20 (M)	Grip strength (kg)	Gait speed ≤0.8 m/s	B and A
		ALM/height^2^ (kg/m^2^)	<16 (F), <27 (M)	Short Physical Performance Battery ≤8 points	
		<5.5 (F), <7.0 (M)	Chair stand		
			>15 s/five rises	Timed Up & Go test ≥20 s	
				400-m walk ≥6 min or fail	
AWGS ^(10)^	2019	ALM/height^2^ (kg/m^2^)	Grip strength (kg)	Gait speed <1.0 m/s	(B and/or C) and A
		DEXA: ≤5.4 (F), ≤7.0 (M)	<18 (F), <28 (M)	Chair stand ≥12 s/five rises	
		BIA: ≤5.7 (F), ≤7.0 (M)		Short Physical Performance Battery ≤9 points	
SDOC ^(11)^	2020	-	Grip strength (kg)	Gait speed <0.8 m/s	B and C
			<20 (F), <35.5 (M)		

ALM, appendicular lean mass; AWGS, Asian Working Group for Sarcopenia; BIA, bioelectric impedance analysis; BMI, body mass index; DEXA, dual-energy X-ray absorptiometry; ESPEN-SIG, European Society for Clinical Nutrition and Metabolism Special Interest Group; EWGSOP, European Working Group on Sarcopenia in Older People; FNIH, Foundation for the National Institutes of Health; IWGS, International Working Group on Sarcopenia; SCWD, Society for Sarcopenia, Cachexia, and Wasting Disorders; SDOC, Sarcopenia Definition and Outcomes Consortium.

To prevent sarcopenia, an efficient mass screening method should be established. Physical performance tests require extensive resources, such as longer test time and larger test space. In addition, the variations in test results can be caused by the participant’s condition, education effect in repeated measures, and skill levels of technicians, compared with muscle mass measurement. Thus, physical performance tests may not be feasible for mass screening. The EWGSOP recommended using the SARC-F questionnaire for case finding in their revised definition of sarcopenia ^(9)^. However, SARC-F and other screening questionnaires have low sensitivity and high specificity, suggesting that many true cases may be missed ^(12)^. Contrarily, muscle mass measurements can be feasible, objective, and time-saving tests with high accuracy for sarcopenia mass screening. In this review, we summarize the measurement methods and muscle mass indicators to highlight the issues with previous assessments.

## Measurement Methods of Skeletal Muscle Mass

Skeletal muscle mass is measured using various direct and indirect methods. The major direct methods include computed tomography (CT), magnetic resonance imaging (MRI), dual-energy X-ray absorptiometry (DEXA), and skeletal muscle ultrasound. On the other hand, the major indirect methods include bioelectric impedance analysis (BIA) and D_3_-creatine dilution methods. Each measurement method has advantages and disadvantages, which are presented in Table 2.

**Table 2.** Summary of Measurement Methods of Skeletal Muscle Mass.

Measurement methods	Time	Equipment	Cost	Target muscles	Measures	Cutoff	Adverse effect
Direct methods							
Computed tomography (CT)	Short	Fixed	High	Specific muscle	Cross-sectional area	No	High-level radiation
Magnetic resonance imaging (MRI)	Long	Fixed	High	Whole-body, components, specific muscle	Cross-sectional area	No	-
Dual-energy X-ray absorptiometry (DEXA)	Short	Fixed	Low	Whole-body, components	ALM	Yes	Low-level radiation
Skeletal muscle ultrasound	Short	Portable	Low	Specific muscle	Muscle thickness	No	-
					Muscle thickness		
Indirect methods							
Bioelectric impedance analysis (BIA)	Short	Portable	Low	Whole-body, components	ASM	Yes	-
D_3_-creatine dilution method	Long	-	High	Whole-body	Creatine pool size	No	-

ALM, appendicular lean mass; ASM, appendicular skeletal muscle mass.

### Computed tomography

CT is an assessment method that highly distinguishes skeletal muscle from other components, including bone and connective tissues; it is frequently used as the gold standard measurement method for muscle mass ^(13)^. The advantage of CT is its ability to simultaneously measure both the skeletal muscle area (quantity) and density (quality), which reflects intramuscular adiposity links to muscle function. However, it requires the use of large equipment and exposes patients to high-level radiation. Radiation exposure increases the difficulty of using CT for the whole-body measurement of skeletal muscle mass among healthy individuals. However, existing CT images for other purposes can be reanalyzed in a clinical setting for skeletal muscle assessment. The prevalence of sarcopenia and its impact on adverse clinical outcomes have been investigated using CT among patients undergoing routine CT imaging, particularly those with cancer ^(14), (15), (16)^. Generally, the skeletal muscle is assessed *via* CT at the 3^rd^ lumbar vertebra level ^(17)^ but is dependent on the routine CT imaging range, such as the cervical vertebra for head and neck cancers or thoracic vertebra for lung cancer ^(18)^.

### Magnetic resonance imaging

MRI is another high-resolution method for skeletal muscle assessment and is considered a gold standard ^(13)^. Because MRI can capture several weighted images, it enables a more thorough examination of muscle quantity and quality than CT. Furthermore, MRI has no radiation exposure, giving it an advantage over CT. However, MRI requires participants to remain still for a longer period during imaging. High cost and the lack of a reference value for diagnosing sarcopenia are also important limitations of MRI.

### Dual-energy X-ray absorptiometry

DEXA is the most commonly used measurement method for skeletal muscle mass ^(19)^. Compared with CT, MRI, and the four-compartment model, DEXA has a stronger and more concurrent validity (correlation coefficient: r > 0.91) ^(13)^. By emitting two different energy X-rays, DEXA measures lean mass in the whole body and body components as the subtraction of bone and fat. Skeletal muscle and connective tissue make mass ^(20)^. Therefore, DEXA cannot directly determine skeletal muscle mass or assess muscle quality as CT can. However, DEXA has significantly lower radiation exposure than CT but has limited portability for skeletal muscle mass measurement compared with portable devices.

### Skeletal muscle ultrasound

Skeletal muscle ultrasound is an inexpensive method for bedside muscle assessment that has several benefits, including no radiation exposure, low cost, and portability. It can assess muscle thickness, cross-sectional area, echo intensity, pennation angle, and fascicle length. However, the accuracy and comparability of skeletal muscle ultrasound measures could vary between technicians; guidelines have been published to standardize the measurement technique ^(21)^. It is impractical to sequence whole body or appendicular muscle mass using skeletal muscle ultrasound due to the long sequence time. Thus, muscle mass prediction formulas have been developed using selected muscle thickness measured by skeletal muscle ultrasound and validated compared with DEXA ^(22), (23), (24)^ and MRI ^(25)^. Skeletal muscle ultrasound is a reliable and validated method for muscle mass measurement in the diagnosis of sarcopenia ^(26), (27)^.

### Bioelectrical impedance analysis

BIA is an indirect measurement method for muscle mass; it measures the impedance of body components using a combination of electrode positions and electric currents with single or multiple frequencies, as skeletal muscle and fat mass have different impedances ^(28)^. BIA has no radiation exposure and is feasible for use in any setting owing to its portability. However, it has the following limitations: some conditions, such as edema, increases measurement errors, and implant medical devices are not allowed for testing. The validity and reliability in estimating muscle mass are high for multiple-frequency BIA and moderate for single-frequency BIA ^(13)^. To improve validity, many equations have been proposed that modele age, sex, and anthropometric variables ^(29)^. BIA also measures phase angle, which may reflect muscle quality and function ^(30)^. Lower phase angle has been associated with lower muscle functions ^(31)^.

### Creatine-(methyl-d_3_) (D_3_-creatine) dilution method

The D_3_-creatine dilution method indirectly assesses the skeletal muscle mass by linking it to total-body creatine pool size ^(32)^. The skeletal muscle is the largest creatine pool in the whole body. After creatine is absorbed from the intestine, it is diluted in the creatine pool and excreted in urine by the kidney. Then, creatine isotope (D_3_-creatine) diluted in the creatine pool and total-body creatine pool can be estimated by measuring the urinary concentration ratio of D_3_-creatine to total creatine. D_3_-creatine dilution relies on four assumptions to accurately measure the total-body creatine pool. First, D_3_-creatine is absorbed without any loss. Second, it is completely diluted in the creatine pool before excretion. Third, it is distributed only in the skeletal muscle. Fourth, its distribution is consistent in all skeletal muscles and is not altered by any condition. However, because the last three assumptions are not completely true, errors in the measurement of skeletal muscle mass occur when using the D_3_-creatine dilution method. Although muscle mass measured by the D_3_-creatine dilution method is strongly correlated with the MRI- and DEXA-measured muscle mass, the D_3_-creatine dilution method measures muscle mass smaller than DEXA ^(33)^. Furthermore, lower and decreased muscle mass measured by the D_3_-creatine dilution method is associated with worsened physical function, and a higher risk of physical disability and mortality, but DEXA-measured muscle mass adjusted for height is not ^(34), (35), (36), (37)^.

## Muscle Mass Indicators

Muscle mass can be measured using three indicators: cross-sectional area, thickness, and mass weight. To accurately assess muscle mass, various indicators have been created. However, no single indicator has a consensus with the definition of sarcopenia. Different muscle mass indicators can be used depending on the definitions of sarcopenia, including ALM, appendicular skeletal muscle mass (ASM), relative skeletal muscle mass (%ALM), SMI, and ALM/BMI.

### ALM and ASM

ALM and ASM are the crude measures of lean and muscle mass, respectively. ALM is the lean mass weight of all upper and lower limbs measured by DEXA. Meanwhile, ASM is the skeletal muscle mass in all upper and lower limbs estimated by BIA. Owing to their similarities and shared uses, ALM is used as a common term for ALM and ASM in this review. Research has demonstrated that lower ALM is associated with a higher risk of mortality but not disability in US men ^(38)^. In US women, no association between ALM and mortality and disability was observed. It is noteworthy that the association of ALM with disability and mortality varies across different populations ^(39)^. Therefore, ALM was added to the revised sarcopenia definition of EWGSOP as a muscle loss measure ^(9)^. In that definition, the cutoff value of ALM was below 20 kg for men and below 15 kg for women. Nevertheless, it is noteworthy that ALM has a strong positive correlation with height and weight ^(40)^.

### Adjustment for anthropometry in muscle mass assessment

There are several approaches to adjusting for anthropometry in muscle mass assessment. The three major parameters are SMI, %ALM, and ALM/BMI. SMI was first proposed by Baumgartner RN et al. in 1998 ^(41)^. Due to the strong correlation between muscle mass and height, SMI is measured as ALM divided by height squared, which is the best variable to minimize the correlation between ALM and height. This indicator still has a moderate positive correlation with weight and BMI ^(42)^. Although SMI is most frequently accepted as a muscle loss indicator in the definition of sarcopenia, lower SMI was associated with higher mortality but not with disability among men and women in the US ^(38)^ and Japan ^(39)^. Conversely, an inverse association of SMI was apparent with the risk of disability and mortality among men and women in another Japanese cohort ^(43)^.

Focusing on weight components, %ALM and ALM/BMI were also used to assess muscle quantities. %ALM was proposed by Janssen I et al. to adjust for nonskeletal muscle mass ^(8)^, calculated as ALM divided by the whole-body weight. %ALM is a muscle loss component of sarcopenia defined by the ESPEN Special Interest Group ^(2)^. Because whole-body weight is associated with height and fat mass, %ALM can be partially adjusted for height-related components and fat mass. However, a modest inverse correlation was observed between %ALM and weight and BMI ^(42)^. To date, the association between %ALM and long-term health outcomes has not been well examined. In a Japanese study, no association was reported after age adjustment ^(39)^.

The FNIH sarcopenia project recommended using ALM/BMI as a muscle loss measure ^(5)^. Because lower SMI was not strongly associated with physical function ^(44), (45)^, they determined which muscle mass indicator strongly predicts slow gait speed (<0.8 m/s) and found that ALM was the best muscle mass indicator predicting slow gait speed in men but not in women ^(46)^. As ALM/BMI consistently predicted worse physical performance in both sexes, they accepted ALM/BMI as muscle loss indicator in their sarcopenia definition ^(5)^. However, ALM/BMI was not associated with the risk of disability and mortality among Japanese individuals ^(39)^.

In addition, the residual of linear regression modeling was analyzed using ALM as the dependent variable and height and total fat mass as the explanatory variables ^(44)^. A positive association between the residual and leg function was observed in both men and women, whereas a positive association between the residual and SMI was seen only in men. However, the reference formula may not be generalizable to other populations. Further investigation is required to create the ideal reference formula.

### Perspectives

All muscle indicators strongly correlate with body size; thus, we used SMI, %ALM, and ALM/BMI to adjust for height, weight, or BMI. However, even after adjusting for these variables, the muscle indicators remained moderately correlated with body size. Therefore, the use of muscle indicators as part of the sarcopenia diagnosis criteria may have biased the diagnosed sarcopenia from the true sarcopenia. Furthermore, the residual correlation between adjusted muscle indicators and body size may interfere with the appropriate assessment of muscle loss. Further research on muscle mass assessment is necessary to establish a clear definition of sarcopenia.

## Article Information

This article is based on the study, which received the Medical Research Encouragement Prize of The Japan Medical Association in 2022.

### Conflicts of Interest

None

### Sources of Funding

This work was supported by JSPS KAKENHI grant number 22H03351.

### Author Contributions

IM contributed to the search of previous publications and writing of the whole manuscript.

### Approval by Institutional Review Board (IRB)

Because this is not a research of human beings, approval from the institutional review board is not required.
